# Reply: Health Literacy or Care Redesign in Atrial Fibrillation?

**DOI:** 10.1016/j.jacadv.2026.103091

**Published:** 2026-07-31

**Authors:** Timothy J.H. Lathlean, Rajiv Mahajan

**Affiliations:** a School of Medicine, College of Health, Adelaide University, South Australia, Australia; b South Australian Health and Medical Research Institute (SAHMRI), South Australia, Australia; c The Commonwealth Science and Industrial Research Organisation (CSIRO), Precision Health, Adelaide, Australia; d Lyell McEwin Hospital, Northern Adelaide Local Health Network, South Australia, Australia

We thank Dr Karakasis for their letter and thoughtful perspective on health literacy, related interventions and care redesign. Dr Karakasis has raised 2 important questions.

The first question is whether the interventions in the meta-analysis be considered care-redesign or health literacy interventions. The concept of health literacy continues to evolve beyond patient education, now encompassing both individual and organizational dimensions and incorporates several integral aspects of care redesign.^1^ Also, a meta-analysis can only synthesize the questions already posed in the included studies. Acknowledging this limitation, our meta-analysis followed a well-established definition of health literacy,^2^ and investigated whether health literacy interventions led to patient action, behavior change, and improved health outcomes based on the concept coined by Nutbeam. We are confident that this approach maintains construct validity while summarizing current interventions and contextualizing traditional and evolving definitions of health literacy. Dr Karakasis asserts that interventions including educational videos, decision aids, reminder systems, telemonitoring tools, and clinician-facing software, although increasing understanding, can also act through distinct behavioral, organizational, and therapeutic mechanisms. The author argues that these interventions do not directly impact health literacy and may more accurately be considered forms of care redesign. This aligns with the conclusion of our meta-analysis that health literacy interventions extend beyond patient education. In practice, operational health literacy requires care redesign; accordingly, interventions that empower patients with atrial fibrillation (AF) knowledge and self-management skills are most effectively embedded within system-level care redesign.

The second question concerns the apparent paradox between improvements in AF knowledge and self-efficacy and reductions in hospitalization and mortality, with Dr Karakasis suggesting that care redesign may account for the observed mortality benefit. However, even if these interventions are classified as care redesign, the paradox remains and points to a measurement gap, whereby standard behavioral indicators used in the included studies may not capture meaningful changes. As also highlighted in our manuscript, tools utilized to measure health literacy and self-management have limitations and certain behavioral changes may not be captured by the patient-reported outcome measures (PROMs) instruments utilized in the studies. For example, the included studies predated PROM instruments that systematically assess knowledge of AF risk factors and prompted us to develop a new PROMs to capture this critical element.^3^^,^^4^ An another example in the action plan for AF episodes, which reduce emergency visits and hospitalization, and may represent both patient education and care redesign, were not considered in assessing patient actions due to challenges in carrying out a meta-analysis. They along with unmeasured behavioral changes may have played a role in the paradox between mortality benefit and AF knowledge, self-efficacy, and patient activation as measured by the PROMs utilized in the studies.^4^ Our meta-analysis also acknowledges the changing landscape of health literacy intervention and incorporation at the system level with care redesign. We agree that both in conjunction may have contributed to reductions in hospitalization and mortality.

Dr Karakasis points out that future syntheses should aim to further conceptually disentangle the confusion surrounding health literacy interventions and care redesign strategies. The 2 concepts are rather aligned and their benefits difficult to separate, as redesign initiatives may also represent organizational health literacy interventions that reduce complexity and cognitive burden for patients. We also approached the concept of health literacy from the perspective of precision or personalized health,^4^ not only involving monitoring and collection of data but also personalized decision-making and interventions, addressing both the foundational and contemporary conceptualizations of health literacy. Future efforts should aim to more closely align these concepts to encourage research, policies, and programs needed to eliminate health disparities, promote health equity, and achieve health literacy, thereby improving the health and well-being.
